# Entropy as a High-Level Feature for XAI-Based Early Plant Stress Detection

**DOI:** 10.3390/e24111597

**Published:** 2022-11-03

**Authors:** Maxim Lysov, Irina Maximova, Evgeny Vasiliev, Alexandra Getmanskaya, Vadim Turlapov

**Affiliations:** Department of Mathematical Software and Supercomputing Technologies, Lobachevsky University, 603950 Nizhny Novgorod, Russia

**Keywords:** explainable artificial intelligence, high-level explainable feature, entropy, plant stress, early diagnosis

## Abstract

This article is devoted to searching for high-level explainable features that can remain explainable for a wide class of objects or phenomena and become an integral part of explainable AI (XAI). The present study involved a 25-day experiment on early diagnosis of wheat stress using drought stress as an example. The state of the plants was periodically monitored via thermal infrared (TIR) and hyperspectral image (HSI) cameras. A single-layer perceptron (SLP)-based classifier was used as the main instrument in the XAI study. To provide explainability of the SLP input, the direct HSI was replaced by images of six popular vegetation indices and three HSI channels (R_630_, G_550_, and B_480_; referred to as indices), along with the TIR image. Furthermore, in the explainability analysis, each of the 10 images was replaced by its 6 statistical features: min, max, mean, std, max–min, and the entropy. For the SLP output explainability, seven output neurons corresponding to the key states of the plants were chosen. The inner layer of the SLP was constructed using 15 neurons, including 10 corresponding to the indices and 5 reserved neurons. The classification possibilities of all 60 features and 10 indices of the SLP classifier were studied. Study result: Entropy is the earliest high-level stress feature for all indices; entropy and an entropy-like feature (max–min) paired with one of the other statistical features can provide, for most indices, 100% accuracy (or near 100%), serving as an integral part of XAI.

## 1. Introduction

Explainability of artificial intelligence (AI) results is increasingly considered a necessary property. An arsenal of universal methods for increasing the explainability and interpretability of neural network solutions has been developed. This process was stimulated by early articles exploring the explainability and interpretability predictions of AI results, such as [1,2]. The most effective solutions to this problem were identified as analysis of variance or sensitivity analysis, which is well-known in mathematics and used in traditional areas of science and technology [3]; Fisher’s fundamental criterion for separability in high-dimensional space [4]; and linear discriminant analysis [5].

Subsequently, additional methods were proposed, which remain popular today, such as LIME [6], based on the idea of local linear separability, as well as learning important features through propagating activation differences (DeepLIFT) [7,8]. The most popular method, SHAP, generalized a number of previous methods, and the DeepSHAP method based on DeepLIFT [9] was also introduced. An approach [10] developing the idea of hierarchical interpretability based on a multilevel feature pyramid also proved to be methodically useful for AI applications. Selvaraju, Cogswell, et al. [11] also made a significant contribution to the development of a visual approach to the explainability of deep learning model results.

An important direction for XAI is the estimation of the effective local dimensionality of the feature space. Dimensionality estimation is a classical problem solved by applying the PCA method in a local area of the n-dimensional space under study. To date, methods have been proposed to estimate the global topology of the n-dimensional feature space based on graph models that are interesting for XAI, such as [12]. A library was made available, providing the Scikit-learn API to evaluate global and local intrinsic dimensionality, as well as a possibility of working with benchmark datasets published in the literature [13].

A series of works has been devoted to explainability in graph neural networks (GNN) has appeared, as reviewed in [14]. According to [12], software can be used to extract real-life data for GNN training. GNNExplainer was introduced in 2019 [15] as the first general, model-agnostic approach to GNN-based models, although without a global understanding of predictions. In 2020, PGExplainer [16] was proposed, providing a global understanding of predictions made by GNNs. PGMExplainer was also introduced in 2020 [17], which is based on perturbation of the original graph to eliminate unimportant variables from input–output data, employing explanation Bayesian networks as the last step. GraphMask was introduced in 2021 [18]; it is similar to PGExpaliner and can provide a global understanding of a trained GNN model, in addition to providing relevant paths by using a different mask for each layer.

In the field of explainable AI (XAI), the review by Linardatos et al. [19] deserves special attention; the authors systematized the results of the development of XAI to date. We agree with the assertion made by Linardatos et al. [19] that there remain aspects of explainable AI to be explored, including the lack of performance aside, with considerable potential to unlock in the coming years, which has motivated us to further pursue the development of XAI.

Of considerable interest in the field of XAI is the search for explainable features that are simple and fundamental enough to remain explainable for a wide class of objects and phenomena. Our interest in such explainable features was inspired by a recent article [20] on the zero-shot learning method based on a high-level feature vector. Such a vector ensures the minimization of the dataset required for training, as well as the transfer of training results from one dataset to another, in which some of the categories are missing (or not visible). As an example of high-level features, the article considers high-dimensional spectral curves of HSI pixels.

In the present study, we searched for high-level features for the detection of wheat plant stress state. With the detection of plant stress as the application task, we employed vegetation indices that are widely used in smart agriculture. These indices are calculated based on the original data of multispectral images (MSI), especially on hyperspectral images (HSI), owing to their high-frequency resolution. The most popular vegetation index is NDVI (normalized difference vegetation index); many of its analogs have been used, in previous research, such as GNDVI, CGL, SIPI, GI, etc. [21]. The use of TIR sensors is the most commonly applied method by biologists for the early detection of stress, i.e., early enough to eliminate stress without crop loss. TIR sensors are able to detect plant stress at an early stage based on a slight increase in leaf temperature (by 0.2 °C). TIR leaf images, like HSI channels, are grayscale images.

Artificial intelligence methods are widely applied in smart farming [22,23,24]—mostly deep learning methods. However, the most relevant property of currently applied AI models is the explainability of decisions, which is the main property of explainable AI (XAI). In the field of XAI, approaches have been developed that turn the problem of data dimensionality into an exploitable feature [4], offering easy-to-train decision correctors that can be additionally trained during operation [7].

A successful attempt to create a simple, easily configurable, and efficient XAI network was described in [25]. An XAI-based classifier and regressor, which are simple and easily configurable as part of the user task, were built based on a single-layer perceptron (SLP). However, the decision was largely tied to a specific experiment involving plant drought in the presence of a reference (control group).

Phuong, Dao, et al. [26] conducted a study on early diagnosis of the plant drought stress based on HSI data by means of classical ML, including multilayer perceptron (MLP), considerably overlapping with the conditions of our application task; therefore, we will compare the results of the present study with those reported by Phuong, Dao, et al.

## 2. Materials and Methods

### 2.1. Materials

We conducted an experiment to monitor the drought stress of wheat plants for 25 days under biolab conditions, recording the state of plants from a distance 1 m every 2–3 days [25]. Plants were observed in 3 boxes of 30 pots with 15–20 plants in each pot; 15 pots on the left side of the box were watered, 15 plants on the right side of the box were not watered. The state of the plants was regularly recorded during the experiment at an angle of 90° to the surface using three cameras (sensors): a Specim IQ hyperspectral (HSI) camera (range: 400–1000 nm, spectral resolution: 7 nm, channels: 204; 512 × 512 pix), a Testo 885-2 thermal infrared (TIR) camera (320 × 240 pix), and high-resolution RGB camera (5184 × 3456 pix). The total image volume was 72.2 GB, mainly comprising HSIs. TIR sensors were chosen to directly record the leaf temperature, an increase in which is the earliest feature of a stress condition. HSIs were used primarily as a source for multiple vegetation indices that control the presence and the state of the green mass.

The differences between non-irrigated and irrigated plants in temperature (according to TIR images) and water loss (%, via plant weighing) were recorded on the 1st, 3rd, 6th, 8th, 10th, 12th, 14th, 16th, 19th, 22th, and 25th day of the experiment. The following key events and changes in the state of plants were recorded and compared with those of control plants: (1) an increase in the average temperature of plants by 0.2 degrees after 5 days and (2) the beginning of water loss by the plant after 11 days (about 8% of the water volume). The former is the earliest evidence of drought stress, which happens without water loss and visible changes in the green mass. Detection of plant stress before the onset of water loss is a criterion of “early” detection. After 18 days or somewhat later, we observed a depletion of the plant’s compensatory function, as manifested by a break in the line of monotonic temperature increase.

At the end of the experiment, we compiled data that can be considered a time series, although we opted to consider the problem time-context-free.

### 2.2. The Use of Entropy and Max–Min Features as Universal, Explainable, and High-Level

All HSI-based indices and TIR images were collected as grayscale images, which could be characterized at the preprocessing stage by their histogram with 4 standard statistical features {max, min, mean, std}, supplemented with max–min and entropy.

The idea of using entropy and max–min as universal high-level attributes is based on the fact that entropy is an objective and universal measure for changes in the internal state of complex natural objects and their sets. We considered the important or key states observed in the plants under abiotic drought stress during the experimental period. We can observes the following key states: (1) the initial, essentially homogeneous state of the plants (1st day); (2) if on the 1st day, the plants have not yet formed sufficiently and continue to actively grow and bush, then it resulting in some increase in homogeneity, as in our case (3rd day); (3) an increase in state diversity due to uneven entry into a state of stress resulting from heterogeneous soil moisture (6th day); (4) a non-uniform entry into the state of real loss of moisture by the body of the plant and the beginning of drying (12–19th days); (5) a predominance of withered plants and a further reduction in the diversity of states (25th day). An example of a parallel monotonic process on a certain key range is an increase in plant temperature or an increase in the red leaf color component.

Entropy, as an objective reflection of an object state, can be easy calculated for plants using the histogram of the image pixels belonging to a plant as object of interest. The histogram width (max–min), together with the entropy, can also be used as an explainable feature of the plant state.

**Figure A1:**
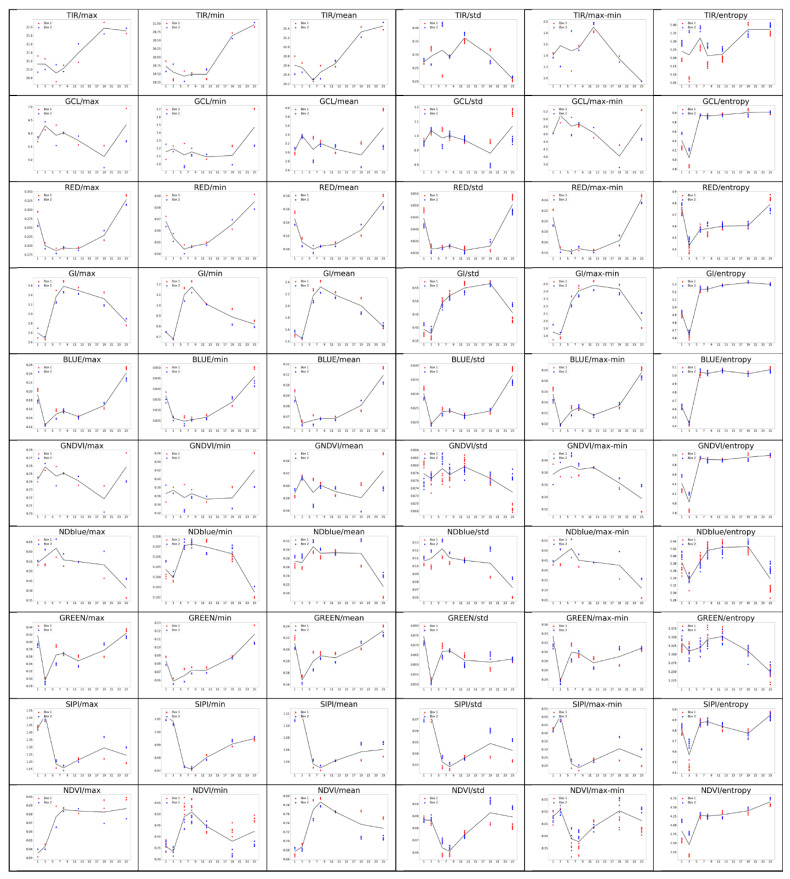
Plots of feature values on key days for all indices.

Graphs of dependence of entropy and max-min feature on key days (see Figure A1, Appendix A) indicate a close and at the same time explainable connection of their values with each key state of plants for all 10 indices. This means that the “high-level” property of the entropy and max-min is confirmed as well.

### 2.3. XAI-Based Classifier Description

**Figure 1:**
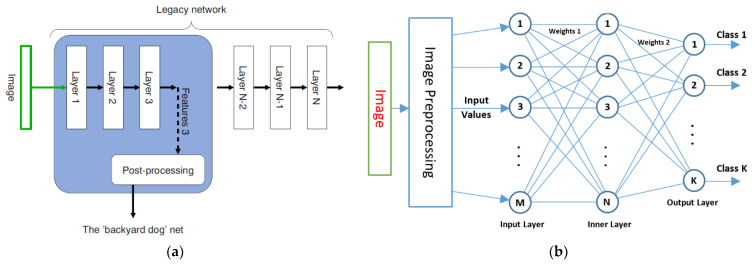
Comparison of the designs of two neural networks: (**a**) the ‘backyard dog’ network from a given legacy network (courtesy of the authors [28] Figure 5); (**b**) the structure of our SLP classifier as an example of the ‘backyard dog’ net implementation for independent use.

In the construction of XAI-based classifier and regressor, which are simple and easily configurable as part of the user task, we followed the example described in [27], implementing the idea of [28]; however, we used the ‘Backyard Dog’ function as the main function of the network, which we implemented via SLP (Figure 1).

To determine *N*, first assume a separate thermal IR channel (TIR), a series of indices, which can be calculated using HSI channel images commonly used in smart agriculture: NDVI, GNDVI, GCL, SIPI, and GI [20]. In addition, consider the capabilities of the 3 visible HSI channels (R_630_, G_550_, and B_480_) as analogs of the red, green, and blue channels of the regular color image. Red, green, and blue channels are of interest for smart farming owing to their high resolution and low price and can be captured using an RGB camera. However, it is generally accepted that they cannot provide sufficient accuracy for the early diagnosis of plant stress. The NDblue index is specially constructed for plant mask building. Hereafter, all 10 objects are collectively referred to as indices. As a result, *I* (=10) indices were accepted for the study, and the number of neurons in the inner layer was chosen as *N = I* + *A*, where *A* is the number of additional neurons reserved to increase accuracy and decrease calculation time. As a result, the maximum number of inputs (*M*) and the maximum number of weights (*Nw*) are expressed as:

*M* = *N* × *h* = 10 × 6 = 60, *Nw = M* × *N + N* × *K* = 60 × 15 + 15 × 7 = 1005 (1)

Our SLP classifier should provide early detection of plant stress in the absence of a clearly defined standard of a stress-free plant in the detected area.

An important condition for the successful application of computational experiments is the correct separation of plant pixels from the background under conditions of the changing state of the plant, soil, and other background objects. The plant mask can be built on both the traditionally used NDVI and other indices that are sensitive to chlorophyll. In connection with the study of the channels of a color image, it is possible to build a mask on the values of the visible range or even on the RGB values. Therefore, mNDblue was used as a base index, which is intended for high-resolution plant leaf images [20]:

mNDblue = −(ρλ − ρ450)/(ρ850 + ρ450) λϵ{530,570,675,730}. (2)

In the present study, we used mNDblue with two modifications: (1) we used λ = 550 for compatibility with the G_550_ hyperspectral channel, and (2) we changed the normalization of Formula (2) to a constant threshold value (Th) for all plant states in the masking process. The result, NDblue, is expressed as:

NDblue = (G550 − B450)/(max(G550 − B450) − min(G550 − B450)); or(3)

NDGB = (G − B)/(max(G − B) − min(G − B))(4)

where G and B are RGB channels.

**Figure 2:**
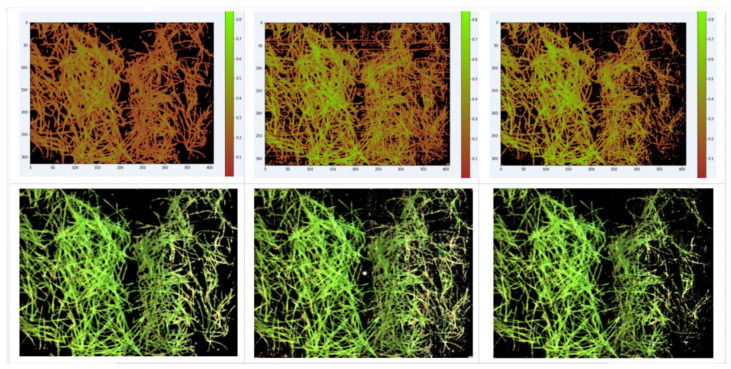
Upper row—day 25 images defining the mask, from left to right: 1st—normalized NDblue index in pseudocolor scale for threshold=0.1; 2nd and 3rd—normalized NDVI index in pseudocolor for the thresholds of 0.15 and 0.25, respectively. Bottom row—images generated by applying upper row masks to three HSI channels (R_630_, G_550_, and B_480_).

Option (3) is for HSI, and Option (4) is for the visible range. Computational experiments were carried out to select the type of the plant mask formation index between traditional NDVI and NDblue, as well as to select the threshold value. The results for one of the days are shown in Figure 2. The NDblue index (3) was chosen, and a threshold Th > 0.1 was set for the plant mask according to experimental results.

The NDblue variant with a threshold of 0.1 was chosen as the most suitable index, preserving the integrity of the plant without capturing background pixels and losing pixels inside the leaf when the state of the plant changes. This selection was made on all key days. Figure 2 shows day 25, when the most visually noticeable changes occurred. The normalization adopted in formula (3) ensures that the threshold is constant for all days.

### 2.4. Exploration Methods

Using the SLP classifier (Figure 1b), in the present study, we aimed to solve the following tasks: (1) determine which components of the input feature vector are the best for early plant stress detection and sufficient for SLP classifier training; (2) determine the role of the high-level features of entropy and max–min in early stress detection; (3) determine whether the construction of an XAI SLP classifier is adequate to solve the problem.

To achieve these goals, we studied early plant stress detection with respect to all 60 the features in the following order: (1) for each feature separately, (2) for the feature pairs within each index; (3) for each index separately using all 6 features; (4) for combinations of indices, excluding TIR, in pairs, triplets, fours, and fives. Each of the tasks was solved for two cases—a short monitoring range (12 days) and a long monitoring range (25 days)—to investigate the differences in stress detection in these two time ranges for smart agriculture applications and to explore their possible co-application.

We found that is was possible to reduce the share of data used to train the classifier to 10–20% of the plants’ mask area without losing classification quality.

The accuracy calculation was organized as an average accuracy calculation over 50 or 100 trials of the stress detection training procedure, generating starting weights for each trial according to the “Kalming” variant of the Kalming He distributions [29].

Before calculating our 6 classification features from histograms, we executed a denoising preprocessing procedure. To this end, we excluded a few percentiles of pixel values from the top and bottom of the histogram. For noisier TIR images, the exclusion of 5 percentiles from the top and 1 percentile from the bottom is sufficient, whereas for HSI-based indices, 1 percentile needs to be removed from both sides to eliminates the main part of the noise and increase the robustness of the features. A special XAI neural network tool was constructed to distinguish and study the 7 key stress states of plants, from no stress to deep stress.

## 3. Results

### 3.1. Significance of Each Feature from the Complete Feature Vector

**Figure 3:**
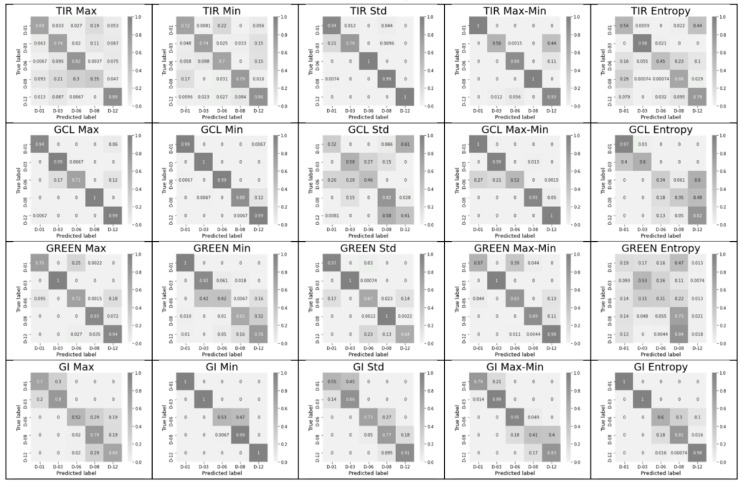
SLP classifier training with one scalar feature. Period: 12 days. D-00—key days with their numbers: 01, 03, 06, 08, 12. Confusion matrices for each scalar feature. The following features are shown in the columns: max, min, std, max–min, and entropy; the most interesting 4 of 10 indices (TIR, GCL, Green, and GI) are shown in the rows.

The significance of each feature for each of the key days is shown in Figure 3 in the form of a confusion matrix.

**Figure 4:**
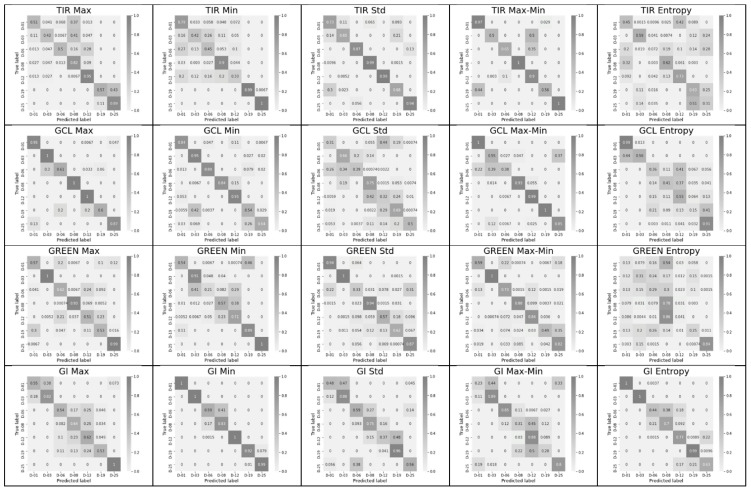
SLP classifier training with one scalar feature. Period: 25 days. D-00—key days with their numbers: 01, 03, 06, 08, 12, 19, 25. Confusion matrices for each scalar feature. The following features are shown in the columns: max, min, std, max–min, and entropy; the most interesting 4 of 10 indices (TIR, GCL, Green, and GI) are shown in the rows.

The significance of each feature for each of the key days is shown in Figure 4 in the form of a confusion matrix for the period of days 1–25.

For a short period (12 days, Figure 3), TIR shows the best diagonality for two features (std and max–min), GCL shows the best diagonality for three features (max, min, and max–min), Green shows the best diagonality for the two features (max and max–min), and GI shows the best diagonality for two features (min and entropy). A similar diagonal pattern persists for the period from 1 to 25 days. The green channel (550 nm) was included in Figure 3 and Figure 4 as the center of the visible range. However, the best indices, according to the sum of short- and long-period accuracy, are Blue and GI (Table 1). GCL and TIR are ranked third and fourth, respectively. Table 1 shows the average plant stress classification accuracy over 100 trials for each of the 6 features for all 10 indices. As a rule, the classification ability of a feature for a full period decreases relative to that for a short period. The maximum values for each of the indices are shown in bold for both periods.

For the TIR, the maximum classification accuracy falls on the std feature in both short and long periods. For the NDVI, Blue (480 nm), and GCL the maximum classification accuracy is associated with the max feature. For Red (630 nm) and GI, the maximum classification accuracy is associated with the min feature. For the GNDVI and NDblue indices, the maximum classification accuracy is associated with the max–min feature, and for the Green index, the maximum classification accuracy is associated with the mean feature.

### 3.2. Significance of Feature Pairs within Each Index Separately and of the Entropy or Max–Min Presence in a Pair

**Figure 5:**
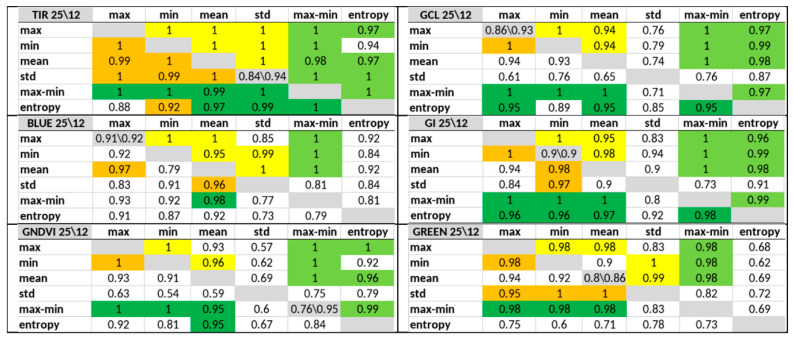
Average accuracy values for the classification of key stress states achieved after training using pairs of features. The accuracy values in the triangle above the gray diagonal correspond to the 12-day period, whereas those under the gray diagonal correspond the 25-day period.

Figure 5 shows the average accuracy of classification of the key stress states achieved for paired combinations of features within each index, indicating the significance of paired combinations for tress detection in each the 12-day (above the gray diagonal) and 25-day (below the diagonal) periods. The most interesting results for 6 (TIR, GCL, Blue, GI, GNDVI, and Green) of the 10 indices are shown. For comparison, the gray diagonal separating the short and long period shows the accuracy for the most significant single feature.

The best pairs (in the range of 0.95 to 1.0) within each index including the entropy or max–min features are indicated by a green background in Figure 5 with (dark green for the 25-day period and light green for the 12-day period). Similar pairs without the participation of the entropy or max–min featured are indicated by yellow in the upper triangular part and orange in the lower triangular part. 

The TIR index has the greatest quantity of such “green” pairs. The other indices also contain a sufficient amount of such pairs for practical use.

SLP classifier training was executed each time using only one of 10 indices and different pairs from of the six features. The paired combinations of features resulted in a classification accuracy equal to or very close to 1.00, with more such cases in the 12-day observational period. The upper average value for pairs inside the NDVI is 0.96. Thus, using the 12-day observation period and any index excluding NDVI at least one pair of features can be found, which provides the early detection of plant stress with an accuracy of 1. The use of the 25-day observation period, an accuracy value of 1.00 can be achieved with only 5 of 10 indices (Figure 5).

Next, we investigated the proportion entropy and max–min features in the pairs required to ensure maximum accuracy. Table 2 shows accuracy values for the 12- and 25-day periods sorted in descending order of their sums.

Table 2 also shows the training time, which characterizes the convergence rate for the training process. If the sums of accuracies are equal, then the table is sorted by the sum of training times. The last row was added to Table 2 specifically to show the best combination of features for NDVI (for comparison with the results reported in [26]). Training for all feature combinations listed in the table was completed in less than 1 s.

More than 60% of the pairs listed in Table 2 include entropy or max–min. Moreover, the use of max–min in a pair is almost always preferable, replacing std in the pair.

The specific unique and high-level role of entropy is demonstrated in the last column of Figure A1 in Appendix A, which shows the entropy graphs for all 10 indices. The same behavior is observed for all indices, as demonstrated by the three start points of the graphs, which characterize the earliest features of plant stress in our experiment. This result supports the universality of entropy as a feature for any index or object, such as the gray-level co-occurrence matrix (GLCM) [30].

### 3.3. Significance of Excluding and Including All Six Features within Each Index and Using a Complete Feature Vector for All Indices Excluding or including TIR

**Figure 6:**
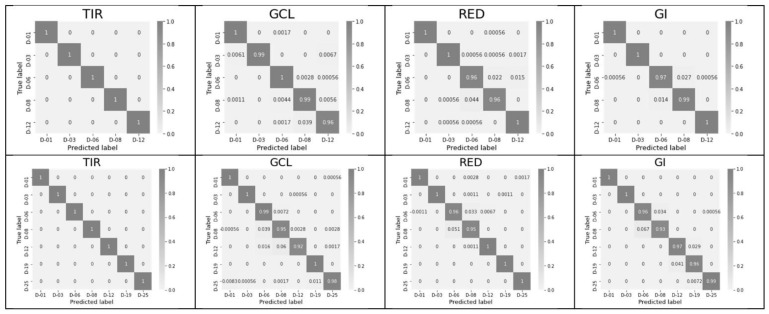
Confusion matrices for the key days after training using all six features per index. Results are shown for four indices (TIR, GCL, Red, and GI). Confusion matrix: true classes are shown on the y axis, and predicted classes are shown on the x axis. D-00 are the key days. The first row corresponds to the 12-day observation period, and the second row corresponds to the 25-day observation period.

Figure 6 shows the confusion matrices for classifications 5 (12-day range) and 7 (25-day range) of the key days using all 6 features within each of 10 indices. Only four examples for the TIR, GCL, Red, and GI indices are shown. The matrices demonstrate a sufficient level of diagonalization and the presence of errors, despite low values.

The average accuracies for the classification of key stress states achieved after training using all 6 features within each of the 10 indices over stress states are shown separately in Table 3. The table includes the accuracies for the 12- and 25-day observation periods, as well as values of the training time in seconds for each case.

SLP classifier training using all six features instead the pairs resulted in decreased detection accuracy. In some cases, such as the NDVI, the decrease is sufficient: from 0.97 for pairs of features to 0.77 for using six features.

Training the SLP classifier immediately on the full vector of features and using the index combinations made it possible to achieve the maximum classification accuracy for all key days for both the 12- and 25-day monitoring ranges (Table 4). 

Here we investigated the possibility of reducing the number of indices necessary for robast classification under TIR exclusion conditions. Table 4 shows that: (1) our SLP classifier can classify all seven stress states of plants with an accuracy of 1 by combining two, three, four, or five indices; (2) the combination of indices results a shorter training time than the use of each index separately; (3) a shorter training time is required for the same combination of indices but for different day ranges when the accuracy is 1 than when a lower accuracy is achieved; and (4) no obvious increase in training time is associated with an increase in the number of indices in the combination.

## 4. Discussion

We compared the results of our experiments with those reported in [26]. The main difference observed was that the plants were in different stages of vegetation at the start of experiment, with a lag in development of the crown (in our case, approximately 3–4 days) but with the same start point in terms of real water losses. Table 5 shows a comparison of our experimental results with those reported in [26]; this comparison highlights two different approaches, classical ML and XAI, with feature selection, combination, and study using XAI methods.

The following questions were resolved in the present study:

(1) An SLP-classifier was built. The classifier structure was adjusted in terms of the number of neurons (*N*) used on the inner layer (according to the number of indices used), the length of the feature vector (*M* = *m* × *N*, where *m* = 1,2,…6), and the number of detected states (key days; *K*).
(2) The classification accuracy of key days was determined individually for each of the 60 possible features and for their pair combinations within an index. Combinations that provide an accuracy of 1 (or near 1), as well as the number of such combinations for each of the indices, were determined, and indices and leaders were established.
(3) We established that the involvement of the full-feature vector does not necessarily result in the maximum accuracy, and a set of at least two features and a few neurons in the inner layer is required to provide a solution to the problem.
(4) We recommend to use the entropy as the main feature, as well as the max–min feature, which determines the number of states for which the entropy is calculated.
(5) It is important to investigate not only the contribution (sensitivity) of individual features but also that of combinations of their minimal numbers inside indices. Therefore, the NDVI achieved the worst performance in index ranking when using all six features (Acc. = 0.77 for 25 days; see Table 3), but the best pair of NDVI features provided an Acc. = 0.97 for the 25-day range (see Table 2).
(6) The use statistical features of the index image instead of the image itself as the MLP input and use of formalized key states as the output ensures the explainability of the SLP classifier as a whole and its high accuracy, representing a valuable XAI research tool.
(7) Increasing the number of indices increases the robustness of the solution. As many as five index combinations may be required to ensure the robustness of solutions for smart farming applications.

The exploration software was implemented via Python 3.8.6. For preprocessing and visualization of TIR images and HSIs, the libraries PySptools, Scikit-image, NumPy, Pandas, Matplotlib, and Cv2 were used. PyTorch and Scikit-learn libraries were used for the neural network models creation, training, and quality estimation. The computer, with an Intel Core i3-8130U, 2.2 GHz, 4 cores, 4 GB, was used as a hardware.

## 5. Conclusions

The results of the study are as follows:

(1) Entropy can be used as a universal high-level explainable feature for classification, in particular for early detection of plant stress. The histogram of the single-channel image pixels belonging to any object of interest is the source for the entropy calculation.
(2) The histogram width determined as max–min also can be used as a high-level explainable feature.
(3) The entropy and the max–min features, in combination with the other histogram statistical features, should be used as the priority high-level input parameters of XAI neural networks (excluding pairs ‘max–min, std’, owing to their high correlation).

XAI networks using feature pairs involving high-level features such as entropy and max–min can be used for the following applications:

− To replace the use of a complete set of statistical features of HSI-based indices;
− To eliminate the need for thermal IR sensors for the early detection of plant stress;
− To significantly reduce the requirements for sensors used in smart farming;
− To eliminate the need for large datasets, energy, computational, and time resources for neural network training; and
− For one-trial correction of AI systems [27].

In this work, the SLP classifier training time was reduced to 0.13–1.0 s.

Additional studies of our XAI approach, including studies on the influence of noise and the robustness of plant stress detection, are planned in the future.

## Figures and Tables

**Table 1:** Significance of each feature for SLP classification in terms of accuracy values.

Index/Feature	Accuracy, 12	Accuracy, 25	Index/Feature	Accuracy, 12	Accuracy, 25
TIR/max	0.70	0.67	RED/max	0.70	0.79
TIR/min	0.76	0.7	RED/min	**0.87**	**0.82**
TIR/mean	0.76	0.67	RED/mean	0.71	0.71
TIR/std	**0.94**	**0.84**	RED/std	0.6	0.54
TIR/max–min	0.88	0.8	RED/max–min	0.63	0.71
TIR/entropy	0.69	0.49	RED/entropy	0.76	0.59
NDVI/max	**0.79**	**0.71**	GCL/max	**0.93**	**0.86**
NDVI/min	0.61	0.50	GCL/min	0.97	0.81
NDVI/mean	0.8	0.68	GCL/mean	0.84	0.74
NDVI/std	0.69	0.65	GCL/std	0.52	0.52
NDVI/max–min	0.55	0.47	GCL/max–min	0.89	0.82
NDVI/entropy	0.55	0.57	GCL/entropy	0.61	0.56
GNDVI/max	0.81	0.72	SIPI/max	0.80	0.74
GNDVI/min	0.91	0.72	SIPI/min	**0.83**	**0.84**
GNDVI/mean	0.82	0.70	SIPI/mean	0.7	0.66
GNDVI/std	0.44	0.37	SIPI/std	0.67	0.66
GNDVI/max–min	**0.95**	**0.76**	SIPI/max–min	0.80	0.72
GNDVI/entropy	0.69	0.55	SIPI/entropy	0.61	0.55
BLUE/max	**0.92**	**0.91**	NDblue/max	0.91	0.71
BLUE/min	0.62	0.64	NDblue/min	0.58	0.51
BLUE/mean	0.79	0.73	NDblue/mean	0.60	0.59
BLUE/std	0.82	0.70	NDblue/std	0.8	0.6
BLUE/max–min	0.8	0.79	NDblue/max–min	**0.93**	**0.74**
BLUE/entropy	0.78	0.6	NDblue/entropy	0.58	0.42
GREEN/max	0.87	0.73	GI/max	0.70	0.67
GREEN/min	0.75	0.69	GI/min	**0.90**	**0.90**
GREEN/mean	**0.86**	**0.8** **0**	GI/mean	0.81	0.79
GREEN/std	0.85	0.75	GI/std	0.76	0.66
GREEN/max–min	0.85	0.76	GI/max–min	0.80	0.61
GREEN/entropy	0.36	0.38	GI/entropy	0.88	0.79

**Table 2:** Average accuracy values after training using feature pairs for 12- and 25-day periods.

Combination of Features	Accuracy, 12 Days	Training Time (12 Days), s	Accuracy, 25 Days	Training Time (25 Days), s
GI/max, min	1	0.19	1	0.31
TIR/max–min, entropy	1	0.20	1	0.34
GCL/min, max–min	1	0.23	1	0.32
GI/min, max–min	1	0.21	1	0.35
GI/max, max–min	1	0.21	1	0.36
GCL/max, min	1	0.29	1	0.32
TIR/std, max–min	1	0.19	1	0.46
GI/mean, max–min	1	0.23	1	0.43
GCL/max, max–min	1	0.31	1	0.38
GCL/mean, max–min	1	0.37	1	0.44
TIR/max, min	1	0.41	1	0.43
TIR/max, max–min	1	0.43	1	0.43
TIR/min, mean	1	0.41	1	0.48
TIR/min, max–min	1	0.40	1	0.50
GNDVI/max, min	1	0.42	1	0.57
GNDVI/max, max–min	1	0.42	1	0.58
GNDVI/min, max–min	1	0.41	1	0.65
TIR/mean, std	1	0.44	1	0.74
TIR/max, std	1	0.59	1	0.62
GREEN/min, std	1	0.55	1	0.79
TIR/std, entropy	1	0.38	0.99	0.60
NDVI/max, mean	0.96	0.8	0.97	0.87

**Table 3:** Average classification accuracy after training using each index separately with six features and the training time for each case.

Index	Accuracy, 12 Days	Training Time (12 Days), s	Accuracy, 25 Days	Training Time (25 Days), s
TIR	1	0.39	1	0.40
GCL	0.99	0.21	0.98	0.34
RED	0.98	0.63	0.99	0.81
GI	0.99	0.23	0.97	0.33
BLUE	0.97	0.66	0.95	0.67
GNDVI	0.96	0.35	0.93	0.54
NDblue	0.91	0.86	0.87	0.91
GREEN	0.82	0.74	0.95	0.89
SIPI	0.85	0.58	0.87	0.87
NDVI	0.79	0.49	0.77	0.58

**Table 4:** The average accuracy after training via the index combinations and the training time.

Combination of Indices	Accuracy (12 Days)	Training Time (12 Days), s	Accuracy (25 Days)	Training Time (25 Days), s
GCL, GI	1	0.15	1	0.17
BLUE, RED	1	0.41	1	0.49
GNDVI, GI	1	0.13	0.99	0.30
GNDVI, RED	1	0.34	0.99	0.53
GNDVI, NDblue	0.97	0.55	1	0.54
GREEN, GCL	0.93	0.49	1	0.42
GNDVI, GREEN	0.9	0.67	1	0.49
GNDVI, RED, GI	1	0.15	1	0.19
GNDVI, BLUE, GI	1	0.15	1	0.21
GNDVI, SIPI, GI	1	0.15	1	0.25
GNDVI, GREEN, GI	1	0.27	1	0.38
GREEN, RED, GI	1	0.48	1	0.45
GNDVI, NDblue, GI	0.99	0.53	1	0.39
GNDVI, BLUE, NDblue	0.99	0.60	1	0.51
GNDVI, GREEN, NDblue	0.97	0.46	1	0.38
GNDVI, GREEN, RED, SIPI	0.99	0.46	1	0.55
GREEN, RED, SIPI, NDblue	1	0.52	0.99	0.67
GREEN, RED, GCL, NDblue	0.99	0.37	0.99	0.3

**Table 5:** The results of two different approaches, classical ML and XAI, for comparison.

Results of [26]	Our Results
A total of three of nine index images (CIRed-edge, mSR705, and SR [21,26]) indicated significant differences after 3 days of water treatment. Most of the indices were sensitive to drought-induced change after 6 days of the water treatment, with the exception of NDVI, ARI, and CCRI.	We used six statistical features instead of the image for each of 10 indices. We considered individual stress detection possibilities for each of 60 features and their pairs for two time ranges (12 and 25 days). All indices were able to detect plant stress states in both intervals with an accuracy 1 or near 1 (0.96). Entropy detected the earliest changes for all used indices, including the NDVI.
Indices cannot be used individually for quality diagnostics on all days. The best result (although not ideal) was achieved by a mixture of indices.	Indices can be employed for quality diagnostics on all days, using some feature pairs. The application of all six features is less productive than the use of pairs. Using a mixture of 2–5 indices guarantees an accuracy 1.
A near-ideal result was achieved by MLP with the use of average HSI signature curves and their derivatives, designated as DNN-Full and DNN-Deriv(atives) at the input.	SLP (practical case of MLP) is sufficient for plant stress classification with an accuracy 1. The employed properties of HSI signature derivatives practically equal to the properties of NDVI.
Owing to the use of hyperspectra as features, it was necessary to train the classifier for each plant type and possible irrigation condition.	Using the entropy and max–min features in a pair with another suitable statistical feature, we obtained the simplest and robust XAI-classifier, independent from plant type, irrigation conditions, temperature, and other external conditions.

## Data Availability

The data used herein were from a 25-day experiment on wheat drought, including records of the state of plants in the control and experimental groups collected every 2–3 days using three types of sensors (HSI, thermal IR, and RGB). The data occupy 72.2 GB and can be obtained from the authors upon reasonable request.
